# Flexible Graphene Paper Modified Using Pt&Pd Alloy Nanoparticles Decorated Nanoporous Gold Support for the Electrochemical Sensing of Small Molecular Biomarkers

**DOI:** 10.3390/bios14040172

**Published:** 2024-04-03

**Authors:** Encheng Sun, Zhenqi Gu, Haoran Li, Xiao Liu, Yuan Li, Fei Xiao

**Affiliations:** 1 Technology Inspection Center of Shengli Oilfield Branch, Sinopec (Shandong) Testing and Evaluation Research Co. Ltd., China Petrochemical Corporation, Dongying 257000, China; sunencheng.slyt@sinopec.com (E.S.); lihaoran819.slyt@sinopec.com (H.L.); liuxiao.1113@163.com (X.L.); liyuan159.slyt@sinopec.com (Y.L.); 2 Sinopec (Shandong) Testing and Evaluation Research Co. Ltd., China Petrochemical Corporation, Dongying 257000, China; 3 Key Laboratory of Material Chemistry for Energy Conversion and Storage, Ministry of Education, School of Chemistry and Chemical Engineering, Huazhong University of Science & Technology, Wuhan 430074, China

**Keywords:** Pt&amp, Pd nanoparticles, nanoporous gold, flexible graphene paper electrode, electrochemical catalysis, small molecular biomarker sensing

## Abstract

The exploration into nanomaterial-based nonenzymatic biosensors with superb performance in terms of good sensitivity and anti-interference ability in disease marker monitoring has always attained undoubted priority in sensing systems. In this work, we report the design and synthesis of a highly active nanocatalyst, i.e., palladium and platinum nanoparticles (Pt&Pd-NPs) decorated ultrathin nanoporous gold (NPG) film, which is modified on a homemade graphene paper (GP) to develop a high-performance freestanding and flexible nanohybrid electrode. Owing to the structural characteristics the robust GP electrode substrate, and high electrochemically catalytic activities and durability of the permeable NPG support and ultrafine and high-density Pt&Pd-NPs on it, the resultant Pt&Pd-NPs–NPG/GP electrode exhibits excellent sensing performance of low detection limitation, high sensitivity and anti-interference capability, good reproducibility and long-term stability for the detection of small molecular biomarkers hydrogen peroxide (H_2_O_2_) and glucose (Glu), and has been applied to the monitoring of H_2_O_2_ in different types of live cells and Glu in body fluids such as urine and fingertip blood, which is of great significance for the clinical diagnosis and prognosis in point-of-care testing.

## 1. Introduction

In the human body, several small molecules such as hydrogen peroxide (H_2_O_2_) and glucose, play an important role in metabolic process and can be recognized as biomarkers. For example, H_2_O_2_, the most common type of reactive oxygen species (ROS), is a byproduct of various oxidase reactions. In organisms, excessive hydrogen peroxide can damage DNA and other biological molecules, which in turn may induce a variety of diseases such as Alzheimer’s disease, myocardial infarction, Parkinson’s disease, cancer, etc. [1,2]. Therefore, the determination of H_2_O_2_ is of practical significance in biological and clinical fields. Meanwhile, the glucose level in blood is a significant biomarker of Diabetes mellitus. In 2019, a total of 463 million people worldwide were diagnosed as diabetic, representing 9.3% of the global adult population. This number is expected to increase to 578 million (10.2%) in 2030 and 700 million (10.9%) in 2045 [3,4]. Therefore, the development of a highly sensitive and selective method for detecting these small molecular biomarkers in real human samples is of great importance in biological and biomedical applications [5,6]. Among the various detection technologies, electrochemical sensors are devices that can detect and measure chemical substances by converting their interaction with electrodes into electrical signals, where chemical reactions between the target substance and the electrodes generate a measurable signal, typically voltage or current [7,8,9]. With the merits of high sensitivity, rapid response times, good long-term stability, ease of miniaturization and cost-effectiveness, electrochemical sensors are highly versatile and applicable in detecting various biomarkers for biomedical monitoring, healthcare, and so on [10,11,12,13].

To date, a variety of nanomaterials have been successfully used to construct electrochemical sensors [14,15,16,17]. Among them, bimetallic nanoparticles (NPs) offer distinct physicochemical properties including high catalytic selectivity, stability and ductility, good tensile strength, and corrosion resistance, which are superior to that of monometallic counterparts [18]. The exceptional catalytic activity of bimetallic systems stems from two primary factors, i.e., the “bifunctional effect” relates to geometric alterations in bimetallic systems versus monometallic ones, and the “ligand or electronic effect” signifies changes in the electronic characteristics of pure metals upon the addition of a second metal component [19]. For example, noble metals such as platinum (Pt) and palladium (Pd) usually exhibit significant catalytic activity for diverse chemical reactions, even at low temperatures [20,21]. While alloys combining Pt (or Pd) demonstrate heightened catalytic effects due to their strong synergistic behavior compared to their monometallic counterparts [22,23,24]. However, the pursuit of suitable supporting materials for achieving efficient electrochemical deposition of alloys remains a formidable challenge. Recently, there has been considerable attention directed toward nanoporous metallic materials that exhibit a distinct continuous network of ligaments with diameters at the nanoscale, showing several unique properties surpassing those of bulk materials [25]. Notably, these nanoporous materials possess a high strength-to-weight ratio, high surface area-to-volume ratio and high ductility during compression [26,27]. Particularly, nanoporous gold (NPG), driven by dealloying processes of gold–silver alloy, showcases an integral morphology with a self-supporting nanostructure [22,23]. It boasts excellent conductivity and a high specific surface area, mitigating issues related to Au-NPs aggregation. Consequently, NPG stands out as an outstanding support material for constructing functionalized layers [28,29,30]. One the other hand, the capability of flexible microelectrode technology can break through the limitations of applicability, miniaturization and spatial resolution, which has made it at the frontier of advancing electrochemical sensors such as implantable and wearable sensor devices for in vivo and in situ real-time detection of various biomarkers in the human body [31]. Given the robust mechanical properties and unique electrical performance of high-surface-area nanocarbon materials, electrocatalysts are commonly supported on these substrates [32]. Significantly, freestanding graphene paper (GP) electrodes demonstrate unique mechanical flexibility, high electrical conductivity, excellent stability, and can facilitate the easy oxidation–reduction of reactants as well [33,34]. Hence, depositing Pd-Pt bimetal on nanoporous gold (NPG) modified graphene paper is a promising strategy for minimizing costs as well as maximizing the electrochemical sensing activity.

**Figure 1:**
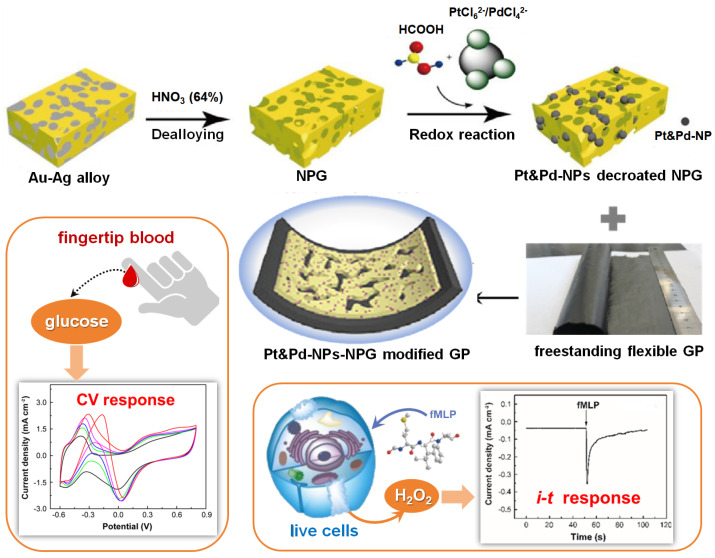
Schematic of the fabrication process of flexible Pt&Pd-NPs–NPG/GP electrodes for electrochemical biosensors towards H_2_O_2_ and Glu.

Herein, we present a new design and facile synthesis of NPG supported alloy nanocatalysts by employing a straightforward single-step method to deposit minute Pt and Pd alloy nanoparticles (Pt&Pd-NPs) onto ultrathin NPG support (Pt&Pd-NPs–NPG), which is modified onto a GP microelectrode for electrochemical sensing of biomarkers including H_2_O_2_ and Glu in real human samples (Figure 1). The synergistic effects between the robust GP electrode substrate, the permeable NPG support, and the deposited ultrafine Pt&Pd-NPs on it give rise to the resultant nanohybrid microelectrode with a large surface area and abundant adsorption sites for target analytes, thus enhancing the electrochemical catalytic activity and the signal-to-noise ratio of miniaturized systems. With an optimal combination of high sensitivity, selectivity, reliability and good biocompatibility, the practical application of the proposed flexible microelectrode was further explored for electrochemical detection of H_2_O_2_ in live cells and Glu in human urine and blood samples, which provides important information for biomedical applications, healthcare monitoring, and so on.

## 2. Experimental

### 2.1. Preparation of Freestanding GP

Graphene oxide (GO) nanosheets were synthesized using the modified Hummers method. The freestanding and flexible GO paper was prepared using the printing method according to our previous work [35]. In detail, the GO suspension with a high concentration of 5~10 mg mL^−1^ was dropped onto the substrate of a commercial cellulose paper and rolled back and forth to enable the GO suspension to uniformly cover the paper. After air-drying, a uniform GO layer coated paper was obtained. The freestanding and flexible graphene paper (GP) was prepared by directly immersing the GO coated paper into HI solution at room temperature for 2 h. During this procedure, the GO layer was peeled off from the cellulose paper to form a freestanding GO paper, which was chemically reduced into reduced GO paper at the same time. The as-obtained GP was repeatedly washed with copious ethanol and deionized water and dried at room temperature for 10 h.

### 2.2. Preparation of Freestanding NPG/GP

Typically, a piece of Au_50_Ag_50_ (weight ratio) alloy membrane (1 cm × 1 cm) was carefully placed on a glass slide and immersed in concentrated nitric acid until it was dealloyed for 30 min in concentrated nitric acid in the dark at 30 °C. After that, the as-obtained freestanding and ultrathin NPG membrane was transferred into the deionized water and rinsed by floating three times. The NPG/GP was prepared by transferring the NPG membrane to the homemade GP (1 cm × 1 cm), followed by dropping 10 μL Nafion solution onto the surface of NPG to make it more stable on the GP substrate.

### 2.3. Preparation of Pt&Pd-NPs–NPG/GP

For the preparation of Pt&Pd-NPs–NPG/GP, chloroplatinic acid hydrate (H_2_PtCl_6_), palladium chlorine acid (H_2_PdCl_4_) and formic acid (HCOOH) were mixed and dispersed in deionized water at room temperature. The total concentration of H_2_PtCl_6_ and H_2_PdCl_4_ was 3 × 10^−4^ M, with a different ratio of H_2_PtCl_6_ to H_2_PdCl_4_, and the concentration of HCOOH was 5 × 10^−3^ M. Then, NPG/GP was carefully transferred to the mixed solution. The Pd&Pt-NPs were synthesized and deposited onto NPG/GP using the redox reaction between H_2_PtCl_6_, H_2_PdCl_4_ and HOOCH at room temperature for 3 h, and washed using deionized water three times. The Pt&Pd–NPG/GP synthesized in precursor solution with the concentration ratios of H_2_PtCl_6_ to H_2_PdCl_4_ of 3:1, 1:1 and 1:3, were denoted as Pt_3_Pd_1_–NPG/GP, Pt_1_Pd_1_–NPG/GP and Pt_1_Pd_3_–NPG/GP. Meanwhile, Pt–NPG/GP and Pd–NPG/GP were synthesized using the same procedure.

### 2.4. Cell Culture

Different live cells including human cervical carcinoma cells (Hela cell), Schwann cells (RSC96) and human umbilical vein endothelial cells (HUVEC cell) were seeded at a density of 10^5^ cells/well into 6-well plates and maintained in a culture medium consisting of Dulbecco’s modified Eagle medium (DMEM) at 37 °C and subcultured every 3 days. The cell number was calculated using a cell counter.

### 2.5. Treatment of Human Body Fluid Samples

The process of obtaining fingertip blood and urine samples for monitoring Glu concentration involves a series of procedural steps. Typically, fingertip blood samples were acquired by cleaning the fingertip area and using a disposable blood lancet or a similar microneedle device to gently puncture the skin surface for blood extraction. For urine samples, participants were required to provide freshly collected urine samples, usually obtained using a urine collection cup. Subsequently, the as-prepared electrochemical sensor was employed to monitor the Glu concentration in these samples (diluted 50-fold in PBS). The sensor induces changes in electrochemical signals in the presence of Glu, indicating variations in the Glu concentration within the samples.

## 3. Results and Discussion

### 3.1. Physical Characterization of the Nanohybrid Paper Electrode

**Figure 2:**
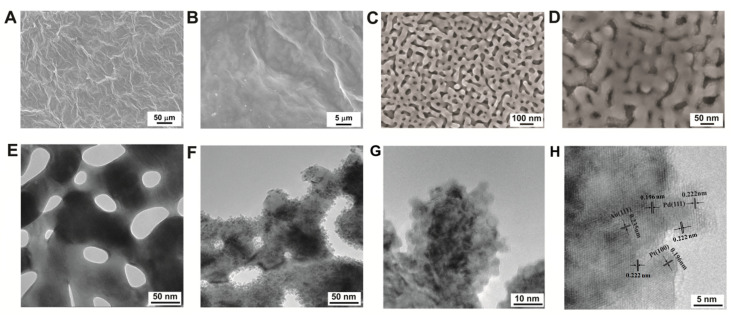
SEM images of (**A**,**B**) GP, (**C**) NPG/GP and (**D**) Pt_3_Pd_1_-NPs–NPG/GP with different magnifications. TEM images of (**E**) NPG and (**F**,**G**) Pt_3_Pd_1_-NPs–NPG with different magnifications. (**H**) HRTEM image of Pt_3_Pd_1_–NPG illustrates the lattice spacings of each element.

Scanning electron microscopy (SEM) was employed to examine the morphology of the freestanding GP. Figure 2A,B reveal that the GP surface exhibits numerous wrinkles, believed to stem from the self-assembly of flexible graphene nanosheets. Notably, SEM images illustrate that the NPG layer deposited on GP comprises interconnected ligaments and numerous nanopore channels, characterized by ligament size ranges of 10~50 nm (Figure 2C). These observations closely corroborate earlier research findings [36]. In-depth analysis of the nanoarchitecture of the NPG membrane was conducted via transmission electron microscopy (TEM), as depicted in Figure 2E. It revealed an ultrathin feature and showcased a characteristic 3D nanoporous arrangement, and its edges were quite smooth, as shown in Figure S1 (Supplementary Materials). For the deposition of Pt&Pd-NPs, the as-obtained NPG support possesses high specific surface area and a controlled effect on the size of Pt&Pd-NPs due to their quantum confinement and edge effects. Figure 2D shows that upon Pt&Pd-NPs deposition, the surface of NPG displays increased roughness. The TEM images of different samples are investigated in detail. It can be observed from Figure S2 that there is an ultra-thin film that entirely covered the surface of NPG for the Pd–NPG sample. As shown in the high-resolution TEM (HR-TEM) image, the film is uniform with a thickness of 2~3 nm. Furthermore, the lattice spacing of 0.235 nm is characteristic of the Au crystalline structure on the NPG support. The interplanar spacing of the film was measured to be 0.222 nm, corresponding to lattice planes of Pd (111). No visible NPs can be seen for Pd–NPG, probably due to the fact that Pd tends to form a dense film rather than NPs on NPG support. Moreover, for Pt_1_Pd_1_–NPG/GP, several seriously agglomerated NPs with a coated film can be seen in Figure S3. With the increase in Pt content in Pt_3_Pd_1_-NPs, it can be observed that the NPG surface is completely covered by a layer of close-packed and well-distributed NPs, with a uniform size distribution of 3 to 5 nm (Figure 2F,G), which are anticipated to have potential for high electrocatalytic activity in electrochemical biosensing applications. The HR-TEM image of Figure 2H shows the excellent crystallinity of Pt_3_Pd_1_-NPs–NPG. The lattice spacing of 0.235 nm is assigned to the NPG support, and the interplanar spacing of Pt_3_Pd_1_-NPs lattice fingers is measured to be 0.196 nm and 0.222 nm, corresponding to well-mixed lattice planes of Pt (100) and Pd (111), respectively. Furthermore, the TEM image of Pt–NPG is similar to that of Pt_3_Pd_1_-NPs, which also reveal the formation of high-density and well-dispersed NPs on the NPG support (Figure S4).

**Figure 3:**
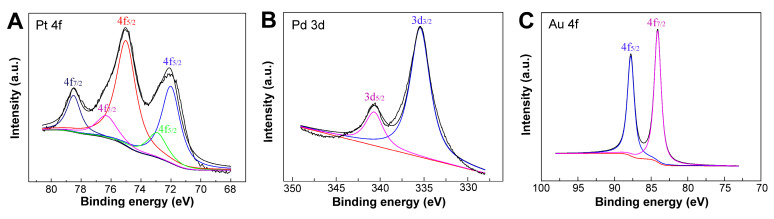
XPS spectra of (**A**) Pt 4f, (**B**) Pd 3d and (**C**) Au 4f of Pt_3_Pd_1_-NPs–NPG/GP.

To determine the chemical composition of Pt3Pd1-NPs–NPG samples, X-ray photoelectron spectroscopy (XPS) analysis was carried out. Figure 3 shows the high-resolution XPS spectra of different element regions. In the Pt 4f spectrum, distinct Pt 4f_7/2_ and Pt 4f_5/2_ peaks are observed, exhibiting a difference in binding energy of 3.33 eV. Deconvolution of these peaks reveals two component peaks in Pt 4f_7/2_ and three in Pt 4f_5/2_. Specifically, the presence of Pt^2+^ ions is indicated by the component peak at 72.9 eV in Pt 4f_7/2_, while the peak at 75.0 eV is attributed to Pt^4+^ ions. As for the Pd 3d spectrum, primary peaks are identified at 335.4 eV and 340.5 eV, corresponding to Pd 3d_5/2_ and Pd 3d_3/2_. Additionally, the Au 4f_7/2_ and Au 4f_5/2_ peaks are observed at binding energies of 84.1 eV and 87.7 eV, respectively, originating from the NPG support. Moreover, a comprehensive assessment of the elemental content reveals an atomic ratio of 20.3% Pt, 6.4% Pd, and 73.3% Au for the Pt_3_Pd_1_-NPs–NPG composition.

### 3.2. Electrochemical Characterization of the Nanohybrid Paper Electrode

The cyclic voltammetry (CV) was employed to perform the electrochemical characterization of different modified GP electrodes in a solution of 0.5 M H_2_SO_4_, covering a potential range of −0.2~1.4 V. The roughness factor (*R_F_*) was calculated using the formula outlined in reference [37]:

(1) $$R_{F}=\frac{A_{r}}{A_{g}}$$

Herein, *A_r_* denotes actual surface area and *A_g_* represents geometric surface area of the nanostructured electrode. For NPG/GP, Pd–NPG/GP, Pt_1_Pd_3_-NPs–NPG/GP, Pt_1_Pd_1_-NPs–NPG/GP, Pt_3_Pd_1_-NPs–NPG/GP and Pt-NPs–NPG/GP, the *A_r_* values were determined theoretically through the reduction peaks of Au oxide species, a method considered more practical compared to utilizing the hydrogen adsorption/desorption peaks of Pt&Pd due to the involvement of quite higher charges [37,38]. The calculation equation is as follows:

(2) $$A_{r}=\frac{\int IdU}{a\cdot v}$$

The constants *a* stand at 435 and 400 μQ cm^−2^ for Pt&Pd-NPs–NPG/GP and NPG/GP, respectively. *I* and *U* values are derived from the CV data, with *v* representing the scan rate during testing. As depicted in Figure S5, the reduction peak potentials for Pt&Pd-NPs–NPG/GP and NPG/GP are aligned closely with the reported values [39,40]. The *A_g_* value of GP is measured to be 0.7 cm^2^, while the *A_r_* value for NPG/GP reaches 3.7 cm^2^. Remarkably, the integration of 3D NPG onto GP has notably augmented the electroactive surface area of GP. Additionally, the subsequent incorporation of densely packed NPs onto NPG has maximized the electroactive surface area of the resulting nanohybrid GO electrode to its fullest potential. The *A_r_* value for Pd–NPG/GP, Pt_1_Pd_3_-NPs–NPG/GP, Pt_1_Pd_1_-NPs–NPG/GP, Pt_3_Pd_1_-NPs–NPG/GP and Pt-NPs–NPG/GP is measured to be 25.5, 32.6, 34.7, 29.8 and 44.2 cm^2^, respectively. Moreover, the calculated *R_F_* for Pt_3_Pd_1_-NPs–NPG/GP stands at the maximal value of 59.98, which is significantly higher than that of NPG (5.25). This discrepancy suggests that the Pt&Pd-NPs–NPG/GP electrocatalyst exhibits enhanced electrocatalytic activity, particularly in selectively accelerating kinetical reactions. Therefore, Pt_3_Pd_1_-NPs–NPG/GP (i.e., Pt&Pd-NPs–NPG/GP) was chosen as the optimal electrocatalyst in the further experiments.

The electrochemical properties of different GP-based electrodes are assessed using electrochemical impedance spectroscopy (EIS). Nyquist plots for GP, NPG/GP and Pt&Pd-NPs–NPG/GP are depicted in Figure S6. The *R*_ct_ values for GP, NPG/GP and Pt&Pd-NPs–NPG/GP are 109 Ω, 93 Ω and 89 Ω, respectively. Evidently, all freestanding GP-based electrodes exhibit remarkably low resistance and swift charge transfer capabilities. These characteristics underscore their potential utility as flexible microelectrodes in diverse electrochemical sensing systems.

### 3.3. Electrochemical Sensing Performances of Pt&Pd-NPs–NPG/GP towards H_2_O_2_

**Figure 4:**
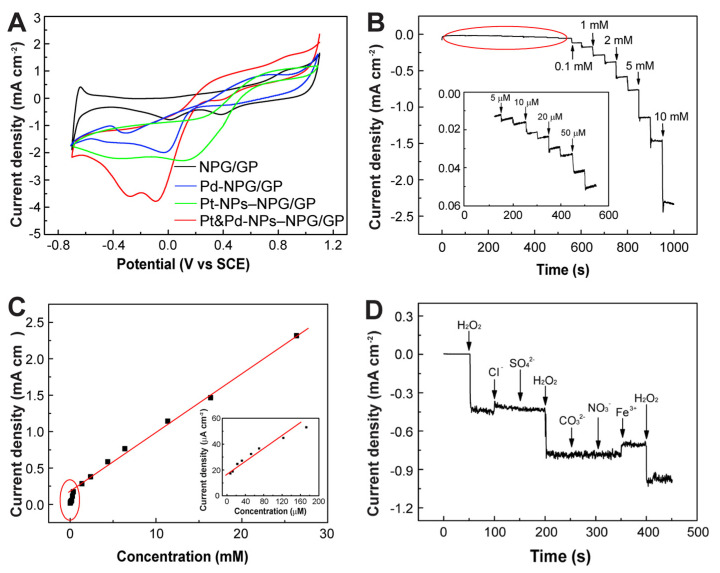
(**A**) CV curves of NPG/GP, Pt-NPs–NPG/GP, Pd-NPs–NPG/GP and Pt&Pd-NPs–NPG/GP in a testing solution of 20 mM H_2_O_2_. Scan rate: 50 mV s^−1^. (**B**) Amperometric responses of Pt&Pd-NPs–NPG/GP to sequential injection of different concentrations of H_2_O_2_ in a stirred PBS solution. Applied potential: −0.1 V. (**C**) Calibration curves of the linear relationship between the current densities and H_2_O_2_ concentrations. (**D**) Amperometric responses to the sequential injection of equal H_2_O_2_ and interference ions.

Since H_2_O_2_ is a crucial small molecular biomarker in cancer studies [41,42], and precise quantitative determination of H_2_O_2_ in biological and environmental samples holds paramount importance [43,44], in this study we conducted an in-depth investigation into the electrochemical catalytic activity of Pt&Pd-NPs–NPG/GP towards H_2_O_2,_ with Pt-NPs–NPG/GP and Pd–NPG/GP as the control samples. Figure 4A exhibits the characteristic cyclic voltammetry (CV) curves obtained from different modified GP electrodes when immersed in a testing solution of PBS (0.1 M, pH 7.4) with 20 mM H_2_O_2_ under optimal detection conditions. The absence of redox peaks of H_2_O_2_ on GP indicates the low electrocatalytic activity of pristine graphene materials towards H_2_O_2_. However, upon NPG modification, a distinct reduced peak around ~0 V emerges in the CV curve of NPG/GP, indicating efficient electrocatalytic reduction of H_2_O_2_ by robust NPG/GP. Furthermore, the CV curve of Pt&Pd-NPs–NPG/GP exhibits a distinct reduced peak of H_2_O_2,_ and reduced peak current density significantly increases. Comparatively, the reduced peak of H_2_O_2_ on Pt-NPs–NPG/GP is quite abroad, and the peak current density is much lower than that on Pt&Pd-NPs–NPG/GP. Meanwhile, the peak current density of H_2_O_2_ on Pd-NPs–NPG/GP is lowest. These results highlight the markedly enhanced electrocatalytic activity of Pt&Pd-NPs–NPG/GP. In this work, the Pt&Pd-NPs–NPG synthesized from the precursor solution with Pt:Pd ratio of 3:1 exhibits high-density and uniform ultra-fine NP structure, rather than the formation of dense film or seriously agglomerated NPs of the control samples. The high-density ultra-fine alloy NPs with well-mixed Pt and Pd atoms can furnish an extensive surface area, abundant active sites, and the synergistic effect between Pt and Pd, which could facilitate the redox reaction of H_2_O_2_ on the electrode.

The amperometric technique was employed to assess the electrochemical sensing capabilities of Pt&Pd-NPs–NPG/GP towards H_2_O_2_, maintaining a fixed potential of −0.1 V. Figure 4B illustrates the current–time (*i-t*) curves of Pt&Pd-NPs–NPG/GP upon continuous addition of H_2_O_2_ into stirred 0.1 M PBS. The sensing system exhibits rapid response kinetics to H_2_O_2_, reaching its maximum steady-state current densities in 2~3 s, demonstrating the rapid absorption, activation and electrochemical reduction of H_2_O_2_ on the electrode. The relationship between current densities and H_2_O_2_ concentration (Figure 4C) reveals a proportional response within a broad concentration range of 0.1 μM to 26.37 mM, demonstrating high sensitivity of 2.37 mA mM^−1^ cm^−2^ and a low detection limit of 0.1 μM at a signal-to-noise ratio of 3. The performances for the detection of H_2_O_2_ using different nanomaterial-based electrochemical sensors are shown in Table S1. The results show that the electrochemical sensing performances of Pt&Pd-NPs–NPG/GP are comparable and superior to that of others and even our previous work [45,46,47,48,49,50,51,52,53], indicating that the proposed work possesses significant potential in various areas of biological and biomedical application.

To evaluate the anti-interference ability, the proposed Pt&Pd-NPs–NPG/GP electrode was tested for the detection of H_2_O_2_ in the presence of possibly interfering ions commonly found in physiological samples. As depicted in Figure 4D, the amperometric responses of the Pt&Pd-NPs–NPG/GP electrode upon sequential additions of 1.0 mM H_2_O_2_ and different ions that commonly existed in human samples were analyzed. Notably, discernible current responses were found exclusively towards H_2_O_2_, and negligible corresponding signals were detected for the other tests. The reproducibility and stability of Pt&Pd-NPs–NPG/GP electrode were further evaluated. Ten identically fabricated electrodes show a relative standard deviation (RSD) of 4.25% in response to 1.0 mM H_2_O_2_. Twenty consecutive measurements using the same electrode result in an RSD of 2.63%, affirming its robust stability for repeated H_2_O_2_ detection. Moreover, the long-term stability investigations reveal that the Pt&Pd-NPs–NPG/GP electrode retains 99.2% and 95.5% of its initial sensitivity after 3 and 30 days, respectively, indicating its high stability.

### 3.4. Real-Time Electrochemical Detection of H_2_O_2_ Secretion by Live Cells

**Figure 5:**
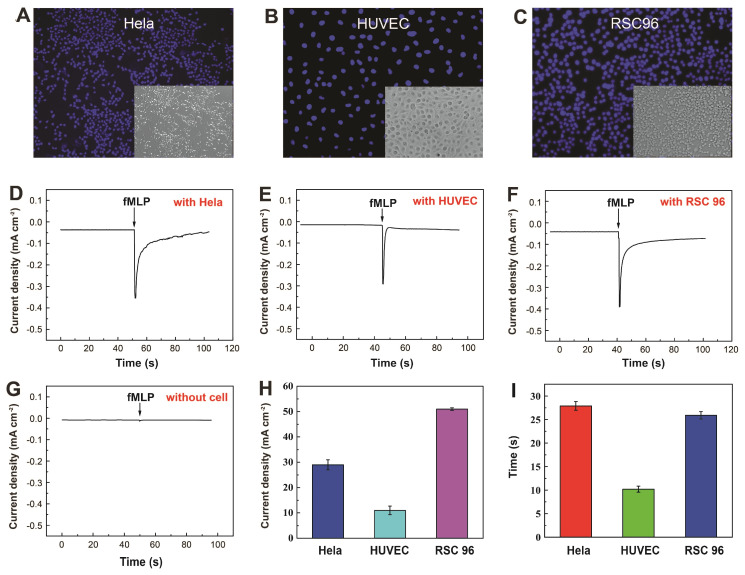
(**A**–**C**) Dark-field and bright-field (inset) images of different cells. (**D**–**F**) Amperometric current responses of the Pt&Pd-NPs–NPG/GP electrode located near different cells under stimulation and (**G**) without cells in PBS solution. Histogram of (**H**) the increased amperometric current densities and (**I**) time to reach the plateau for three types of living cells.

To explore the practical application of the Pt&Pd-NPs–NPG/GP-based biosensor, it was employed to monitor H_2_O_2_ released from living cells, where RSC96 cells, HUVEC cells and Hela cells were selected for real-time monitoring of H_2_O_2_ after stimulation. Microscopic images captured under both bright-field and dark-field conditions demonstrate that following more than 2 h of incubation with the Pt&Pd-NPs–NPG/GP electrode, these cells exhibited sustained viability, and retained their original morphology (Figure 5A–C), highlighting the satisfactory biocompatibility of the Pt&Pd-NPs–NPG/GP electrode.

Then, H_2_O_2_ secretion from living cells at 80% confluency by the administration of N-formyl-methionyl-leucyl-phenylalanine (fMLP), a synthetic peptide known to elicit the secretion of reactive oxygen intermediates [54], was examined. Figure 5D–F shows the amperometric current of the Pt&Pd-NPs–NPG/GP electrode in close proximity to live cells (10^5^ cells/well) within the PBS solution. A remarkable drop in current density was distinct in living cells after the addition of 10 µL of fMLP with concentration of 0.1 mM, subsequently increasing slightly until a plateau was reached. In contrast, there was negligible response upon injection of fMLP without cells (Figure 5G). This suggests the increased responses originated from the H_2_O_2_ secretion from stimulated live cells. Further examination of the current responses from these three cell types yielded intriguing insights. Following equivalent fMLP additions, the amperometric current densities increased by 29 µA cm^−2^ for Hela cells, 11 µA cm^−2^ for HUVEC cells and 51 µA cm^−2^ for RSC96 cells (Figure 5H,I). In addition, the amperometric current response for Hela cells reached the plateau the fastest, followed by RSC96 cells and HUVEC cells (Figure 5I). These findings illustrate the high sensitivity of the proposed method in qualitatively determining H_2_O_2_ released from diverse live cells, which holds great promise for further exploration in clinical diagnosis.

### 3.5. Electrochemical Sensing Performances of Pt&Pd-NPs–NPG/GP towards Glucose

**Figure 6:**
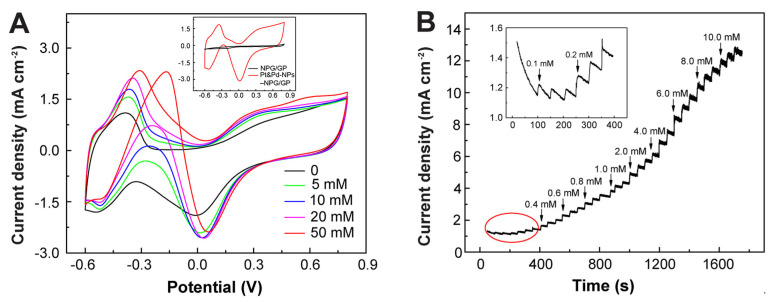
(**A**) CV curves of Pt&Pd-NPs–NPG/GP electrodes for various concentrations of Glu in PBS solution with a scan rate of 50 mV s^−1^. Inset: CV curves of Pt&Pd-NPs–NPG/GP and bare NPG/GP electrodes in a solution of 10 mM Glu. (**B**) *i-t* curve of Pt&Pd-NPs–NPG/GP electrodes for successive additions of different concentrations of Glu.

Diabetes mellitus presents a global public health challenge attributed to metabolic imbalances involving insulin deficiency and hyperglycemia. Deviations in glucose (Glu) concentration beyond the normal range of 4.4~6.6 mM are indicative of this disorder [55]. Therefore, the development of high-performance Glu sensors holds significant importance for diagnosing diabetes mellitus. The electrocatalytic capabilities of Pt&Pd-NPs–NPG/GP have been leveraged for nonenzymatic Glu biosensing, aiming to circumvent the limitations associated with enzymatic Glu sensors, notably insufficient long-term stability and unsatisfactory reproducibility arising from enzyme nature and immobilization activity [56]. CV curves of Pt&Pd-NPs–NPG/GP with different Glu concentrations in testing solution were examined (Figure 6A). Remarkably, distinct anodic peaks related to Glu oxidation and its intermediates are prominently discerned during the positive scan. Conversely, during the cathodic scan at elevated potential ranges, Glu oxidation is hindered by the presence of surface oxides. The reduction of Pt oxide has augmented the active sites accessible for Glu oxidation, leading to a substantial increase in oxidation peak current densities. These findings are consistent with an established mechanism of Glu oxidation on Pt-based electrodes [57]. The redox currents notably escalate with increasing Glu concentration for Pt&Pd-NPs–NPG/GP. In contrast, the CV curves of NPG/GP and GP display nearly absent redox peaks in testing solution containing 10 mM Glu, highlighting the improved electrocatalytic activity attributed to Pt&Pd-NPs on the electrode. Furthermore, the CV curves of 10 mM Glu on Pt-NPs–NPG/GP and Pd–NPG/GP demonstrate the same oxidation peaks of Glu and reduction peaks of Pt/Pd oxide, however, the peak current densities are much lower than that of Pt&Pd-NPs–NPG/GP (Figure S7). This is also due to the significant high surface to volume ratio, abundant active sites and the synergistic effect of alloy NPs, which facilitate the oxidation reaction of Glu on the electrode.

Figure 6B exhibits the *i-t* curve of the Pt&Pd-NPs–NPG/GP electrode upon successive injections of Glu into a PBS solution, while maintaining an applied potential of −0.3 V. The amperometric current response rapidly reaches 95% of its steady-state value within 2~3 s upon each Glu addition. Additionally, a notable linear relationship within the Glu concentration range of 0.1~20.0 mM is achieved, with a high sensitivity of 39.05 μA mM^−1^ cm^−2^ and a detection limit as low as 50 μM (S/N = 3), which are comparable and superior to that of other recently published works including our group’s [58,59,60,61,62,63,64,65,66] (Table S2). Investigations into the potential interference from electroactive compounds commonly found in biological systems, such as dopamine (DA), uric acid (UA), ascorbic acid (AA), cholesterol (Cho) and lactic acid (LA), reveal no interference with the amperometric signals of Glu (Figure S8). The current density changes upon the addition of equal concentrations of these compounds alongside 1.0 mM Glu remain below 5%. This excellent anti-interference performance can be attributed to the low reduction potential of −0.3 V vs. SCE, which inhibits the redox reactions of interfering substances on Pt&Pd-NPs–NPG/GP. Such characteristics render it suitable for sensitive and selective Glu detection in various human body fluid samples.

### 3.6. Electrochemical Detection of Glu in Different Human Body Fluid Samples

Body fluid examination serves as a pivotal tool for diagnosing ailments and monitoring overall health. This type of assessment typically encompasses analyzing blood, urine, cerebrospinal fluid, synovial fluid and other bodily fluids. Through scrutinizing these liquids, clinicians acquire a wealth of information regarding a patient’s health status. For instance, blood tests offer insights into blood cell counts, inflammatory markers, nutritional levels, and indications of various potential diseases. Urine analysis aids in diagnosing renal issues, diabetes and other metabolic concerns. The examination of cerebrospinal fluid assists in detecting neurological disorders, while synovial fluid evaluation aids in diagnosing conditions like arthritis. To illustrate the applicability of the proposed sensor, it has been utilized for Glu detection in other complex biological samples, i.e., fingertip blood and urine. The results are presented in Table 1, which is highly consistent with those obtained using the clinical measurement method. The recoveries of spiked samples fall within the range of 90–109.0%, indicating the high reliability and efficacy of the proposed nonenzymatic electrochemical biosensor based on the Pt&Pd-NPs–NPG/GP electrode. This demonstrates its potential for effectively monitoring various analytes simultaneously in trace amounts within real biological samples.

## 4. Conclusions

In summary, we developed a novel approach for synthesizing alloy nanocatalysts supported by NPG. Our method involved a facile single-step procedure for depositing minute Pd&Pt-NPs onto an ultrathin NPG support, which was then modified onto a freestanding and flexible GP microelectrode. The resulting nanohybrid microelectrode, leveraging the synergistic effects between different components, demonstrates outstanding electrocatalytic activity and favorable biocompatibility, and has been integrated into electrochemical sensors for sensitive and reliable detection of H_2_O_2_ in living cells and Glu in human body fluids. We envision that our proposed strategy in the design of highly active noble metal nanocatalysts and flexible graphene paper electrodes opens a new horizon in the development of high-performance and advanced electrochemical sensing systems, which holds great promise for a wide spectrum of applications in biomedical domains, healthcare monitoring and related fields.

## Figures and Tables

**Table 1:** Results of the Pt&Pd-NPs–NPG/GP-based electrochemical sensing system for the detection of Glu in different human urine and fingertip blood samples.

	Clinical Measurement Method (mM)	The Proposed Method		
		Spiked (mM)	Found (mM)	Recovery (%)
Urine I	0	0	0	/
		0.2	0.18	90
		0.5	0.54	108
Urine II	0	0	0	/
		1	1.09	109
		2	1.95	97.5
Urine III	0	0	0	/
		5	5.17	103.4
		10	9.56	95.6
Fingertip blood I	4.62	0	4.53	98.1
		1	5.67	100.9
		5	10.35	107.6
Fingertip blood II	8.35	0	8.95	107.2
		1	9.64	103.1
		5	14.52	108.8
Fingertip blood II	10.56	0	9.86	93.37
		1	11.97	103.5
		5	16.5	106

## Data Availability

The data presented in this study are available on request from the corresponding author.

## Supplementary Materials

The following supporting information can be downloaded at https://www.mdpi.com/article/10.3390/bios14040172/s1, Figure S1: TEM images of NPG; Figure S2: TEM images of Pd film on NPG support; Figure S3: TEM images of Pt_1_Pd_3_ on NPG support; Figure S4: TEM image of Pt-NPs on NPG support; Figure S5: CV curves of Pt-NPs-NPG/GP, Pt_3_Pd_1_-NPs-NPG/GP, Pt_1_Pd_1_-NPs-NPG/GP, Pt_1_Pd_3_-NPs-NPG/GP and NPG/GP electrodes in 0.5 M H_2_SO_4_ solution. Scan rate: 50 mV s^−1^; Figure S6: EIS Nyquist plots of Pt&Pd-NPs-NPG/GP (a), NPG/GP (b) and GP (c) at open circuit potential with an ac perturbation of 5 mV in the frequency range of 1000 kHz to 0.01 Hz. Inset: equivalent circuit diagram proposed for analysis of the EIS data; Figure S7: CV curves of Pd-NPG/GP, Pt-NPs-NPG/GP and Pt&Pd-NPs-NPG/GP in 0.1 M PBS (pH 7.4) in the presence of 50 mM Glu; Figure S8: Amperometric responses to the consecutive addition of 1 mM Glu and 1 mM interfering species in a stirred PBS solution (0.1 M, pH 7.4); Table S1: Comparison of the performances for the detection of H_2_O_2_ by different nanomaterials-based electrochemical sensors; Table S2: Comparison of the performances for the detection of Glu by different nanomaterials-based electrochemical sensors [49,50,51,52,53,54,55,56,57,58,59,60,61,62,63,64,65,66].
