# Supplementary material for: Flexible Graphene Paper Modified Using Pt&Pd Alloy Nanoparticles Decorated Nanoporous Gold Support for the Electrochemical Sensing of Small Molecular Biomarkers
Source: Biosensors (Basel). 2024 Apr 3;14(4):172. doi: 10.3390/bios14040172 (PMC11048118; doi:10.3390/bios14040172)
Supplement: Supplementary file 1 [file biosensors-14-00172-s001.zip › biosensors-2885990-supplementary.pdf]

Supplementary Materials

Flexible graphene paper electrode modified by Pt&Pd alloy nanoparticles decorated nanoporous gold support for electrochemical sensing of small molecular biomarkers

Encheng Sun[1], Zhenqi Gu[1,*], Haoran Li[1], Xiao Liu[1], Yuan Li[1], Fei Xiao[2,*]

[1]Technology Inspection Center of Shengli Oilfield Branch, Sinopec (Shandong) Testing and Evaluation Research Co. Ltd, China Petrochemical Corporation, Dongying, 257000, P. R. China; sunencheng.slyt@sinopec.com (E.S.); lihaoran819.slyt@sinopec.com (H.L.); liuxiao.1113@163.com (X.L.); liyuan159.slyt@sinopec.com (Y.L.)

[2] Key Laboratory of Material Chemistry for Energy Conversion and Storage, Ministry of Education, School of Chemistry and Chemical Engineering, Huazhong University of Science & Technology, Wuhan, 430074, P. R. China

[*] Correspondence: guzhenqi.slyt@sinopec.com (Z. G.); xiaofei@hust.edu.cn (F. X.)

1. Experimental

1.1. Chemicals and materials

Expanded graphite was purchased from Qingdao Tianheda Graphite Co., Ltd. (Qingdao, China). Potassium permanganate ($KMnO_4$), 2-methylimidazole (Meim), sulfuric acid (H_2SO_4, 98 %), hydrochloric acid (HCl, 36%), nitric acid, hydroiodic acid (HI, 48 %), hydrogen peroxide (H_2O_2, 30 %), glucose (Glu), dopamine (DA), uric acid (UA), ascorbic acid (AA), cysteine (Cys), and glutathione (GSH) were purchased from Sigma Aldrich (Shanghai, China). All chemical reagents were used in the experiments directly without further treatment. All solutions were prepared using deionized water (resistivity: 18.25 MΩ cm^{-1}).

1.2. Characterizations

The morphologies of different nanomaterials were characterized by a field-emission SEM (Nova NanoSEM 450) and field-emission TEM (TECNAI G220 U-Twin microscope, accelerating voltage of 200 kV). XPS measurements were performed on VG ESCALAB 250 spectrometer with monochromatic Al Kα (1486.71 eV) X-ray radiation (15 kV and 10 mA) and hemispherical electron energy

analyzer. The binding energies (BE) were calibrated by setting the measured BE of C 1s to 284.8 eV. For electrochemical testing, Pt&Pd-NPs–NPG/GP electrode, Pt wire electrode and saturated calomel electrode (SCE) have been used as working electrode, counter electrode and reference electrode respectively. The three-electrode system was connected with CHI electrochemical workstation for testing.

2. Results and discussion

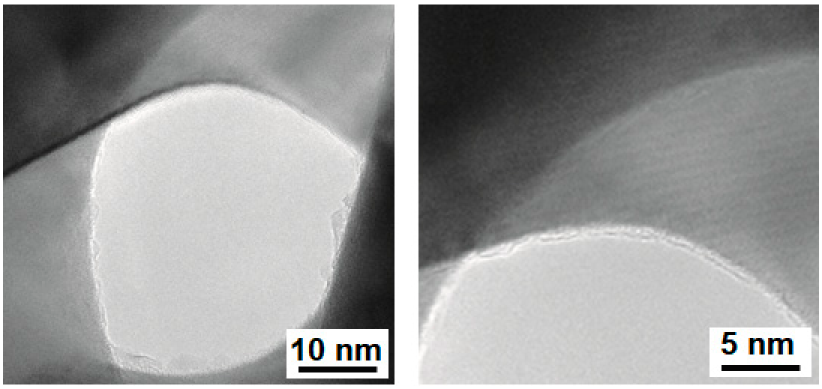

Figure S1. TEM images of NPG.

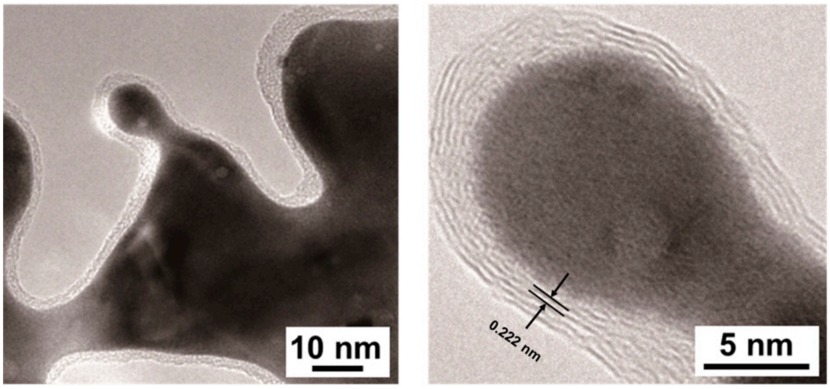

Figure S2. TEM images of Pd film on NPG support.

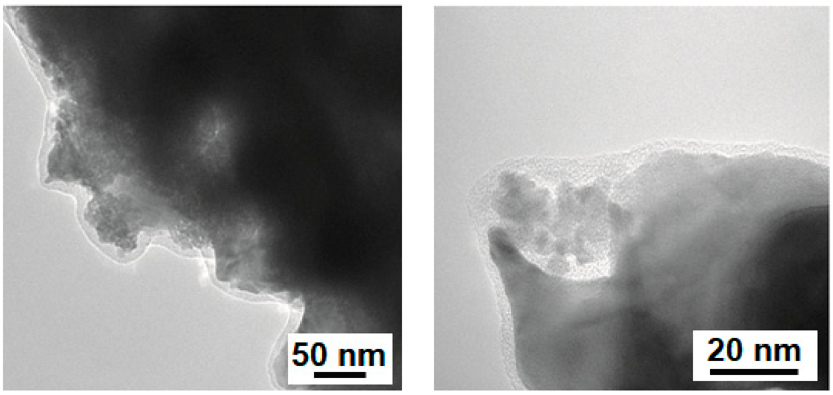

Figure S3. TEM images of Pt_1Pd_3 on NPG support.

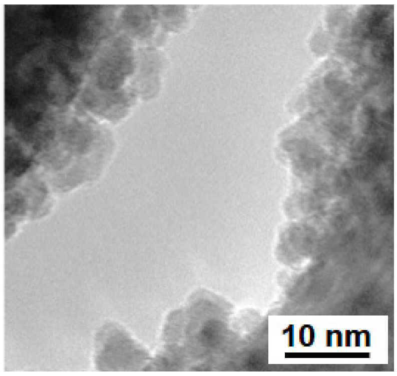

Figure S4. TEM image of Pt-NPs on NPG support.

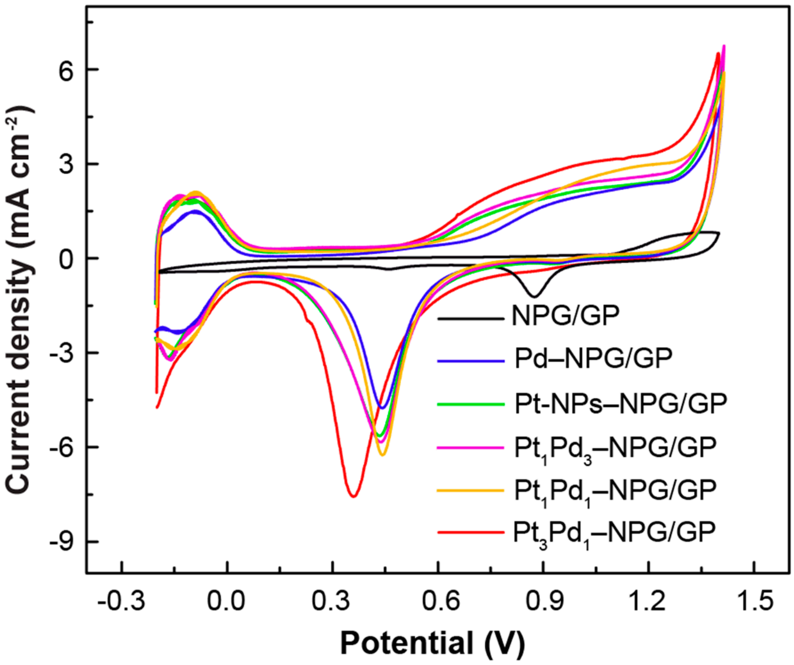

Figure S5. CV curves of Pt-NPs–NPG/GP, Pt_3Pd_1-NPs–NPG/GP, Pt_1Pd_1-NPs–NPG/GP, Pt_1Pd_3-NPs–NPG/GP and NPG/GP electrodes in 0.5 M H_2SO_4 solution. Scan rate: 50 mV s^{-1}.

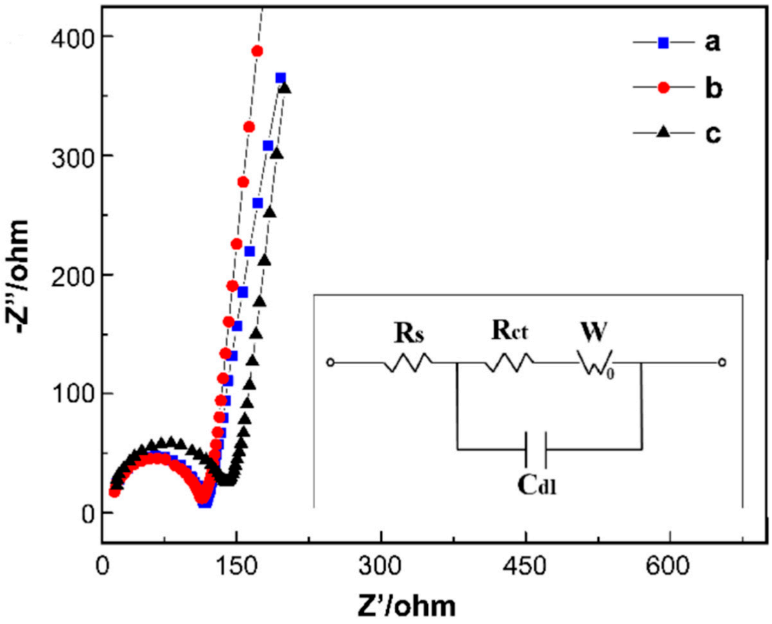

Figure S6. EIS Nyquist plots of Pt&Pd-NPs–NPG/GP (a), NPG/GP (b) and GP (c) at open circuit potential with an ac perturbation of 5 mV in the frequency range of 1000 kHz to 0.01 Hz. Inset: equivalent circuit diagram proposed for analysis of the EIS data.

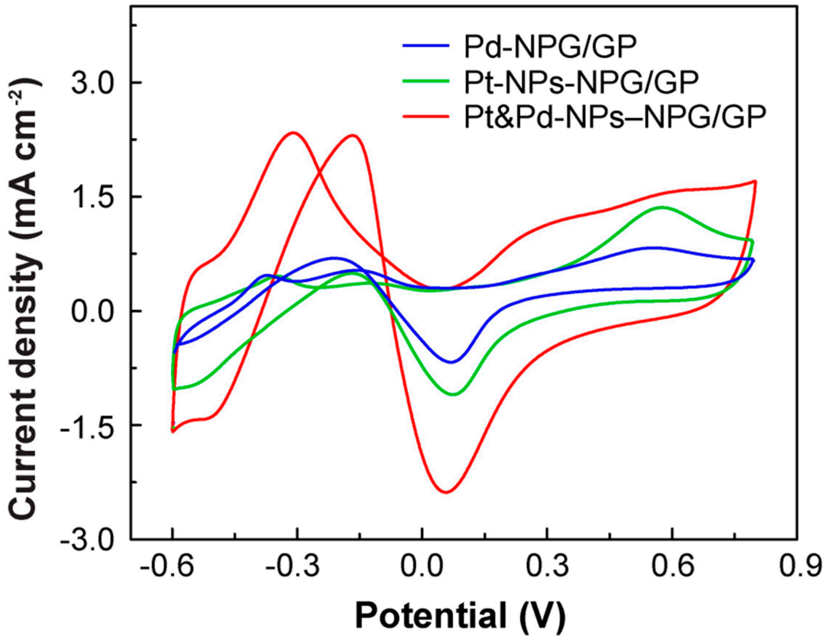

Figure S7. CV curves of Pd–NPG/GP, Pt-NPs–NPG/GP and Pt&Pd-NPs–NPG/GP in 0.1 M PBS (pH 7.4) in the presence of 50 mM Glu.

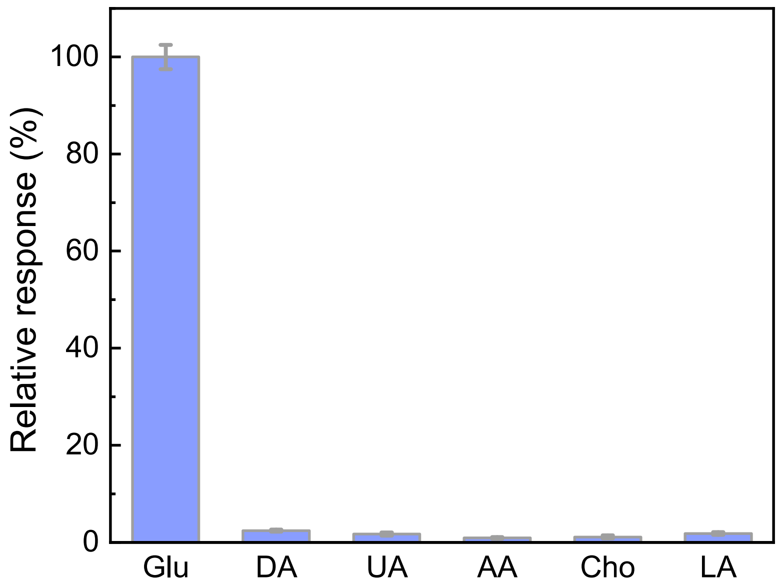

Figure S8. Amperometric responses to the consecutive addition of 1 mM Glu and 1 mM interfering species in a stirred PBS solution (0.1 M, pH 7.4).

Table S1 Comparison of the performances for the detection of H_2O_2 by different nanomaterials-based electrochemical sensors.

Materials	Detection limit	Linear range	Sensitivity	Reference
Pt&Pd-NPs-NPG/GP	0.1 μM	0.1 μM–26.37 mM	2.37 mA mM^{-1} cm^{-2}	This work
Cu-BHT	0.08 μM	0.08 μM–400 μM	257 μA mM^{-1} cm^{-2}	45
ZnO_3-CuO_7	2.4 μM	3 μM–530 μM	1.11 μA $μM^{-1}$ cm^{-2}	46
NBP-CNW-NTAs/CF	0.5 μM	1 μM–15.92 mM	61.8 μA $mM^{-1}cm^{-2}$	47
MNC-900	0.13 μM	1 μM–26 mM	/	48
CuCo-Cu@CoCH	0.97 μM	1 μM–2000 μM	3.29 mA $mM^{-1}cm^{-2}$	49
AuNPs-rGO	6.50 μM	25 μM–3000 μM	0.064 mA mM^{-1} cm^{-2}	50
Au NPs/UiO-66	0.045 μM	0.2 mM–23 mM	0.329 mA mM^{-1} cm^{-2}	51
AuPd-PDA NTs	0.26 μM	1 μM–11.22 mM	0.314 μA mM^{-1} cm^{-2}	52
AuNPs-rGOP papers	2 μM	5 μM–8.6 mM	236.8 μA mM^{-1} cm^{-2}	53

Table S2 Comparison of the performances for the detection of Glu by different nanomaterials-based electrochemical sensors.

Materials	Detection limit	Linear range	Sensitivity	Reference
Pt&Pd-NPs−NPG/GP	50 μM	0.1~20.0 mM	39.05 μA mM^{-1}	This work
CMCNFs-1000/GOx	17.2 μM	0.1~6 mM	2.34 μA mM^{-1}	58
Cu_2O/TiO_2 nanotube	62 μM	3.0~9.0 mM	14.56 μA mM^{-1}	59
Cu_2O/Cu	37 μM	0.05~6.75 mM	62.29 μA mM^{-1}	60
$Ni(OH)_2$/insulin/rGO/Au	5 μM	0.005~10 mM	18.9 μA mM^{-1}	61
Au/Pt-black/Nf	480 μM	1.0~30.0 mM	145.33 μA mM^{-1}	62
ZnO nanorods	1 mM	0.1~13.8 mM	2.97 μA mM^{-1}	63
Pt/CFs	33 μM	0.3~17 mM	2.03 μA mM^{-1}	64
PtPd/nanopores carbon	150 μM	1.5~12 mM	0.11 μA mM^{-1}	65
Au-rGO paper	20 μM	0.1~30 mM	58.54 μA mM^{-1}	66
